# Arabidopsis ETHYLENE RESPONSE FACTOR 8 (ERF8) has dual functions in ABA signaling and immunity

**DOI:** 10.1186/s12870-018-1402-6

**Published:** 2018-09-27

**Authors:** Feng Yi Cao, Thomas A. DeFalco, Wolfgang Moeder, Bo Li, Yunchen Gong, Xiao-Min Liu, Masatoshi Taniguchi, Shelley Lumba, Shigeo Toh, Libo Shan, Brian Ellis, Darrell Desveaux, Keiko Yoshioka

**Affiliations:** 1 Department of Cell and Systems Biology, University of Toronto, 25 Willcocks Street, Toronto, ON M5S 3B2 Canada; 2 Department of Plant Pathology and Microbiology, Institute for Plant Genomics and Biotechnology, Texas A&M University, College Station, TX 77843 USA; 3 Center for the Analysis of Genome Evolution and Function (CAGEF), University of Toronto, 25 Willcocks Street, Toronto, ON M5S 3B2 Canada; 4 Michael Smith Laboratories, University of British Columbia, 2185 East Mall, Vancouver, BC V6T 1Z4 Canada; 5 Present address: Department of Plant and Microbial Biology, University of Zurich, Zollikerstrasse 107, CH-8008 Zurich, Switzerland; 6 Present address: Department of Life Sciences, School of Agriculture, Meiji University, 1-1-1 Higashimita, Tama-ku, Kawasaki, 214-8571 Japan; 7 Present address: Kyoto Research Laboratories, YMC CO., LTD., 59 Yonnotsubo-cho Iwakuraminami, Sakyo-ku, Kyoto, 606-0033 Japan

**Keywords:** ERF, Ethylene response factor, Cell death, Map kinase, MPK4, MPK11, ERF8, MITOGEN-ACTIVATED PROTEIN KINASE, ABA

## Abstract

**Background:**

ETHYLENE RESPONSE FACTOR (ERF) 8 is a member of one of the largest transcription factor families in plants, the APETALA2/ETHYLENE RESPONSIVE FACTOR (AP2/ERF) superfamily. Members of this superfamily have been implicated in a wide variety of processes such as development and environmental stress responses.

**Results:**

In this study we demonstrated that ERF8 is involved in both ABA and immune signaling. ERF8 overexpression induced programmed cell death (PCD) in Arabidopsis and *Nicotiana benthamiana.* This PCD was salicylic acid (SA)-independent, suggesting that ERF8 acts downstream or independent of SA. ERF8-induced PCD was abolished by mutations within the ERF-associated amphiphilic repression (EAR) motif, indicating ERF8 induces cell death through its transcriptional repression activity. Two immunity-related mitogen-activated protein kinases, MITOGEN-ACTIVATED PROTEIN KINASE 4 (MPK4) and MPK11, were identified as ERF8-interacting proteins and directly phosphorylated ERF8 in vitro. Four putative MPK phosphorylation sites were identified in ERF8, one of which (Ser103) was determined to be the predominantly phosphorylated residue in vitro, while mutation of all four putative phosphorylation sites partially suppressed *ERF8*-induced cell death in *N. benthamiana*. Genome-wide transcriptomic analysis and pathogen growth assays confirmed a positive role of ERF8 in mediating immunity, as *ERF8* knockdown or overexpression lines conferred compromised or enhanced resistance against the hemibiotrophic bacterial pathogen *Pseudomonas syringae*, respectively.

**Conclusions:**

Together these data reveal that the ABA-inducible transcriptional repressor ERF8 has dual roles in ABA signaling and pathogen defense, and further highlight the complex influence of ABA on plant-microbe interactions.

**Electronic supplementary material:**

The online version of this article (10.1186/s12870-018-1402-6) contains supplementary material, which is available to authorized users.

## Background

Sophisticated cellular signaling pathways govern plant responses to diverse environmental stimuli. The phytohormones salicylic acid (SA), ethylene, and jasmonic acid (JA), play important roles in plant immunity [1]. In particular, SA plays a well-documented and critical role in promoting defense responses to biotrophic pathogens [2]. While less characterized, defense-related roles for other phytophormones, including auxin, gibberellic acid (GA), and abscisic acid (ABA), have also been documented [1]. ABA, in particular, has been thoroughly characterized as regulating both responses to abiotic stresses, such as drought, salinity, and cold, and developmental processes such as germination [3, 4]; however, recent evidence suggests that ABA also plays a complex role in regulating plant immunity [5].

Immunity-related phenotypes have been previously documented for several ABA-related mutants, including the ABA receptors *PYRABACTIN RESISTANT-LIKE 8* (*PYL8*), *PYL10* and *PYL11*, the protein phosphatase 2C (PP2C) *HYPERSENSITIVE TO ABA1* (*HAB1*), *SNF1-RELATED KINASE 2.6* (*SNRK2.6*), as well as the ABA biosynthesis genes *ABA DEFICIENT 1* (*ABA1*), *ABA2* and *ABA3* [6–8]. The influence of ABA on the outcome of plant-pathogen interaction is also dependent on the pathosystem of study, the method of ABA application, the mode and stage of pathogen infection, and many other factors [9, 10]. Although the molecular mechanisms by which ABA influences immunity are not well understood [5, 9], multiple points of antagonism between ABA and SA signaling have been reported, suggesting that ABA may suppress immunity via suppression of SA signaling [11–14]. While ABA-SA antagonism likely emerges from multiple points of crosstalk [15], empirical study of such points remains minimal. A refined understanding of ABA-SA antagonism can thus provide valuable insight into the balance between abiotic and biotic stress signaling pathways.

Plants are able to defend themselves against most microbes by recognizing conserved microbe- or pathogen-associated molecular patterns (MAMPs or PAMPs, respectively) via a suite of cell-surface pattern recognition receptors (PRRs) [16]. Recognition of MAMPs/PAMPs by their cognate PRRs activates a cascade of intracellular signaling, ultimately leading to pattern-triggered immunity (PTI), which is sufficient to prevent the proliferation of most potential pathogenic microbes [16]. To maintain virulence, some pathogens have evolved effector proteins that perturb host cellular processes and ultimately dampen defense. The hemibiotrophic bacterial pathogen *Pseudomonas syringae*, for example, utilizes a type III secretion system to deliver effectors into the plant cell [17, 18], and phytohormone signaling pathways have been identified as targets of effectors from multiple pathogens [5, 17, 19]. For example, the *P. syringae* effectors AvrPtoB and HopAM1 activate ABA signaling to promote bacterial virulence, although the specific molecular mechanisms involved remain unknown [20, 21]. The outcome of plant-pathogen interactions largely depends on the ability of plants to directly or indirectly recognize effector proteins, as such recognition elicits a secondary layer of defense responses, known as effector-triggered immunity (ETI) [22, 23]. A characteristic ETI response against biotrophic pathogens is the establishment of robust programmed cell death (PCD) at the site of infection, known as the hypersensitive response (HR), which is thought to isolate invading pathogens in the dead cell clusters and prevent systemic infection [24].

Transcription factors are crucial and common elements in signaling pathways involved in abiotic and biotic stresses responses [25–27]. One of the largest transcription factor families in plants, the APETALA2/ETHYLENE RESPONSIVE FACTOR (AP2/ERF) transcription factor superfamily, is characterized by one or more conserved N-terminal ERF DNA binding domain(s). Members of this superfamily have been implicated in a wide variety of processes, including development and environmental stress responses, as well as hormone signaling and pathogen defense [25, 28, 29]. Notably, specific members of group VII, VIII and predominantly group IX within the ERF family have been shown to play a role in Arabidopsis immunity [29–32]. The class II ERF transcriptional repressors, part of group VIII ERFs (subgroup a), are characterized by one of the most abundant repression motif in plants, the ERF-associated amphiphilic repression (EAR) motif -L/FDLNL/F(x)P [33–35]. This small group of EAR motif-containing ERFs has 8 members in Arabidopsis, with limited functional characterization. ERF4 contributes to senescence (together with ERF8) as well as susceptibility to the necrotrophic fungal pathogen *Fusarium oxysporum* [36, 37], ERF9 contributes to resistance against the necrotrophic fungus, *Botrytis cinerea* [38], while ERF7 and ERF11 may be part of the transcriptional repressor complex in ABA or ethylene and GA signaling, respectively [39–41]. Interestingly, one member of this subfamily, ERF8, is part of the complex network of protein-protein interactions that are transcriptionally regulated by ABA [42], and as such is expected to play a role in ABA signaling.

Here, we demonstrated a role of ERF8 in both ABA-mediated responses and immunity. We found that over-expression of ERF8 is sufficient to induce PCD-like cell death, and that ERF8 positively regulates immunity against *P. syringae*. Moreover, ERF8 interacts with and is phosphorylated by two immunity-related mitogen-activated protein (MAP) kinases (MPKs), MPK4 and MPK11. Both MPK4 and MPK11 are activated by bacterial PAMPs [43, 44], while MPK4 has also been demonstrated to be important for many SA-dependent pathogen defense responses [45–47]. RNA-Seq analysis showed ERF8 overexpression leads to transcriptional changes of genes involved in ABA signaling as well as pathogen defense and cell death regulation. Hence, ERF8 represents a potential point of crosstalk between ABA-mediated abiotic stress responses and SA-mediated pathogen defense.

## Results

### ERF8 is a negative regulator of ABA-mediated responses

**Fig. 1:**
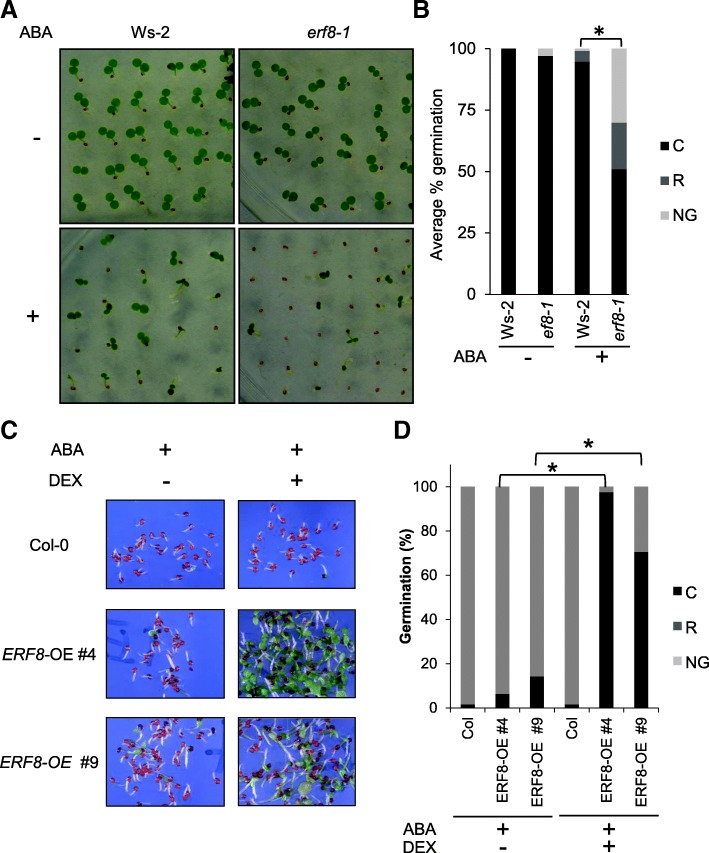
Seeds of *ERF8* knockdown (*erf8–1*) and overexpression lines (*ERF8-OE)* exhibit altered ABA sensitivity*.* Wildtype Ws-2 and *erf8–1* knockdown seeds were sown on 0.5 X MS agar plates with or without 0.8 μM ABA. (**a**) Representative seed populations under 24 h light exposure at 5 days post stratification (dps). (**b**) Average percentage of germination of two replicates at 5 dps. Graph depicts the proportion of seeds that were not germinated (NG), exhibited radicle (R) or cotyledon(s) emergence (C). (**c**) Two independent lines of DEX-inducible *ERF*8-*OE* seeds germinated in the presence of 1 μM ABA with or without 50 nM DEX. (**d**) Two independent lines of DEX-inducible *ERF*8-*OE* seeds were germinated in the presence of 1 μM ABA with or without 50 nM DEX. Average percentage of germination at 14 dps.*p ≤ 0.01

ERF8 has previously been shown to be a component of the transcriptionally-regulated ABA interactome network [42], suggestive of a role in ABA signaling. Furthermore, microarray data indicated an increase in *ERF8* expression during germination (Additional file 1A) [48]. Thus, to examine the role of ERF8 in ABA signaling, ABA-mediated seed germination inhibition assays were performed with *ERF8* gain-of-function and loss-of-function mutant seeds. For these assays we used the confirmed *ERF8* knockdown line FLAG157D10 [36], hereafter referred to as *erf8–1* (Ws-2 background), as well as dexamethasone (DEX)-inducible *ERF8* overexpression lines (*ERF8-OE,* Col-0 background). Over-expression of ERF8 after DEX treatment of these lines was confirmed by Western blot (Additional file 1b). As shown in Fig. 1, *erf8–1* seeds showed increased sensitivity to ABA (Fig. 1a, b) while *ERF8-OE* seeds showed decreased sensitivity (Fig. 1c, d), indicating a negative role of ERF8 in ABA-mediated germination and seedling establishment.

### ERF8 overexpression induces programmed cell death (PCD) in a. Thaliana and N. Benthamiana

**Fig. 2:**
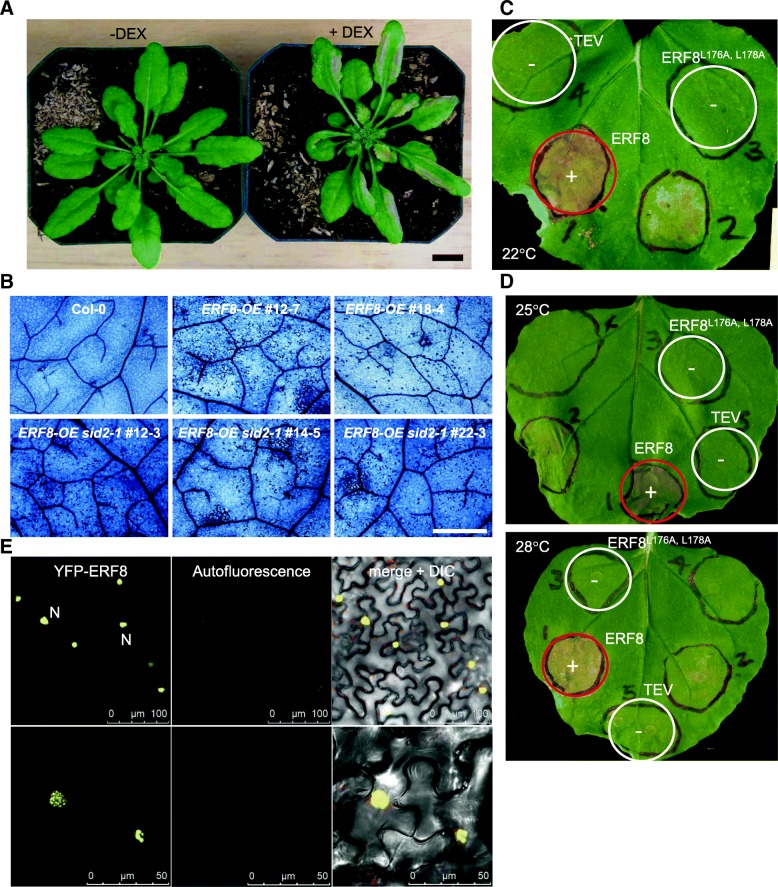
Over-expression of *ERF8* triggers programmed cell death (PCD). (**a**) DEX-inducible overexpression of *ERF8* triggered cell death in transgenic Arabidopsis at 48 h after DEX treatment. Scale bar = 1 cm (**b**) DEX-inducible expression of *ERF8* in Col-0 and *sid2–1* background led to PCD in Arabidopsis. Leaves from Col-0 and various DEX-inducible *ERF8-OE* lines were sprayed with 30 μM DEX and stained with trypan blue solution. Images were taken 2 days after DEX treatment. Scale bar = 1 mm. (**c**) Wildtype ERF8 but not ERF8^L176A, L178A^ elicited strong cell death in *N. benthamiana*. Photo taken 6 days post inoculation (dpi) (+/ red circle = cell death, −/ white circle = no cell death). (**d**) ERF8 overexpression induced cell death in *N. benthamiana* at both 25 °C and 28 °C 3 dpi. (**e**), Transient expression of YFP-tagged *ERF8* in *N. benthamiana* localizes to the nuclei (example nucleus labeled N). Scale bar top = 100 μm, bottom = 50 μm

It was previously reported that constitutive expression of *ERF8* causes lesions in cotyledons and true leaves and associated with a higher rate of premature death before flowering [36]. Thus, we characterized cell death associated with ERF8 in DEX-inducible *ERF8-OE* lines. As shown in Fig. 2, both micro- and macroscopic PCD was clearly observed 2 days post DEX treatment (Fig. 2a, b). Moreover, cell death induction was also observed after *Agrobacterium*-mediated transient overexpression of ERF8 in *Nicotiana benthamiana* 3 days post infiltration (Fig. 2c). HR cell death is a well-described, SA-dependent form of defense-related PCD that is often observed after infection with avirulent biotrophic and hemibiotrophic pathogens [49, 50]. To test the SA-dependency of ERF8-induced cell death, DEX-inducible ERF8 overexpression lines were cross-pollinated with the *salicylic acid induction deficient* 2 (*sid2–1)* mutant. Interestingly, ERF8*-*induced PCD occurred to a similar degree in *sid2–1* as in Col-0 wildtype background plants (Fig. 2b), positioning ERF8 downstream or independent of SA biosynthesis. To corroborate this finding, we next tested the temperature sensitivity of ERF8-induced cell death. Previous studies have shown elevated temperature can supress the development of *resistance* (*R*) gene-mediated HR [51], and many SA-dependent autoimmune mutants such as *suppressor of npr1–1, constitutive 1* (*snc1*), *suppressor of salicylic acid insensitive4* (*ssi4)*, and *constitutive expresser of PR genes22* (*cpr22*) showed suppression of immune responses including HR-like spontaneous cell death under modestly elevated temperature (i.e. 28 °C) [51], indicative of a connection between temperature and HR cell death. ERF8-induced cell death in *N. benthamiana*, however, was unaffected by elevated temperature (28 °C) (Fig. 2d), corroborating that ERF8 likely acts downstream or independent from SA accumulation.

### The EAR motif is required for ERF8-induced cell death

Ogata et al. [52] reported that transient expression of several EAR motif-containing group VIII ERFs including ERF8 induced cell death in *Nicotiana tabacum*. The C-terminal EAR motif has been shown to be crucial for their transcriptional suppressor function [35, 53]. Given the presence of this EAR motif in ERF8, we set out to determine whether the transcriptional repressor activity of ERF8 is required for its function in promoting cell death. When ERF8 was transiently expressed in *N. benthamiana* as a yellow fluorescent protein (YFP)-fusion, we observed clear signals within nuclei, as expected for a transcriptional repressor (Fig. 2e, top). Higher magnification images revealed that ERF8 is not localized evenly in the nucleus but rather in discreet nuclear bodies (Fig. 2f, bottom). A similar pattern has been reported previously for ERF4 and TCP14 TCP (TEOSINTE BRANCHED1, CYCLOIDEA, PROLIFERATING CELL FACTORS 1 and 2) and it has been suggested that these are sites of protein inactivation and degradation [54, 55].

**Fig. 3:**
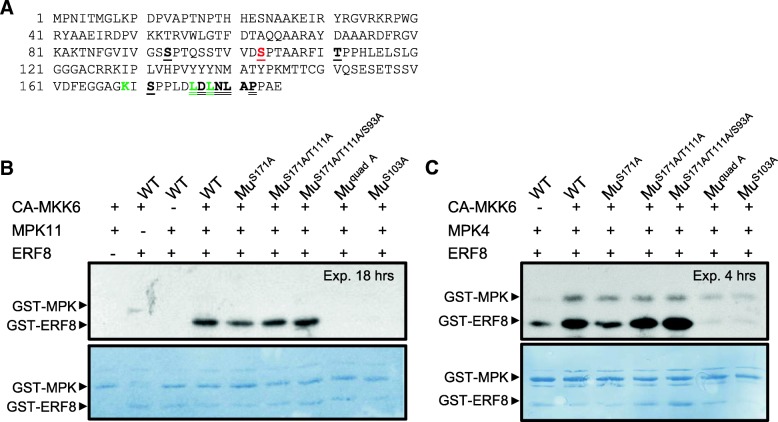
In vitro phosphorylation of ERF8 by MPKs. (**a**) Amino acid sequence of ERF8. Bold and underlined are the 4 putative phosphorylation sites (S93, S103, T111, S171), double-underlined is the amphiphilic repression (EAR) motif. Green residues are the putative kinase docking site (Hamel et al. 2011). (**b, c)** Autoradiographs (top panel) and Coomassie R-250 stained nitrocellulose (bottom panel) are shown. All proteins used were GST-tagged. In reactions containing MPK11 (**b**) or MPK4 (**c**) and GST-ERF8; the presence of WT or mutant (Mu) protein is indicated above the image. Four potential phosphorylation sites were changed to alanine via site-directed mutagenesis: S171A, T111A, S103A, and S93A (quad A = quadruple mutant). CA-MKK6 is a constitutive active version of MAP kinase kinase 6, which phosphorylates MPK4 and MPK11

A double leucine to alanine mutation within the EAR motif (L/FDLNL/F(x)P [56, 57], was introduced to disrupt the function of the EAR motif (ERF8^L176A/L178A^) (Fig. 3a). Although the L176A/L178A mutation did not affect the nuclear localization of ERF8-YFP (Additional file 2a), it abolished the ability of HA-tagged ERF8 to induce cell death (Fig. 2c, d). Western blotting confirmed that ERF8^L176A/L178A^ expression was not reduced compared to wild type ERF8 (Additional file 2B), and that ERF8^L176A/L178A^ ultimately accumulated to higher levels than wildtype ERF8 due to the onset of cell death induced by the latter. Interestingly, it had been reported that the EAR motif of the poplar transcriptional repressor, PtiZFP1, partially overlaps with a bipartite MAP kinase docking site, (R/K)_n_X_n_(LXL), which is also found in the C terminus of group VIIIa ERFs [58]. To distinguish whether transcription suppression through the EAR motif or the potential kinase docking/interacting function of ERF8 is important for cell death induction, the ability for ERF8 and ERF8^L176A/L178A^ to interact with MAP kinases was examined. For this analysis, MPK11 was used, as it had previously been identified as an ERF8 interacting protein in yeast two-hybrid (Y2H) assays (Additional file 3A). We were able to validate this interaction between ERF8 and MPK11 as well as the related kinase MPK4 *in planta* using bimolecular fluorescence complementation (BiFC) assays in *N. benthamiana* (Additional file 3B). Both ERF8 and ERF8^L176A/L178A^ clearly interacted with MPK4 and MPK11 in the nucleus, indicating that the L176A/L178A double mutation specifically disrupted the EAR motif and not the putative MPK docking site. Thus, transcription suppression activity is crucial for ERF8-induced cell death.

### MPK4 and 11 phosphorylate ERF8

Given the *in planta* interactions between ERF8 and MPK4 or MPK11, we tested the ability of these MPKs to directly phosphorylate ERF8 in vitro using recombinantly-expressed GST-fusion proteins. GST-tagged MPK4 or MPK11 were both able to phosphorylate GST-ERF8 in ^32^P-γ-ATP kinase assays in the presence of a constitutively-active MAP kinase kinase (MKK), CA-MKK6 [59] (Fig. 3b, c). Four potential MPK phosphorylation sites, Ser93, Ser103, Thr111 and Ser171 (Fig. 3a), were predicted within ERF8 in silico using PhosPhAt [60]. Single, double, triple and quadruple phospho-dead (Ser/Thr to Ala) mutants were generated using site-directed mutagenesis and tested for phosphorylation by MPKs in vitro. The ERF8^S171A/T111A/S93A^ triple site mutant and other mutant variants with Ser103 unchanged were phosphorylated identically from wildtype ERF8 (Fig. 3). However, both the quadruple mutant (ERF8^S171A/T111A/S93A/S103A^ = ERF8^quad A^) or the ERF8^S103A^ single mutant were no longer phosphorylated by either MPK4 or MPK11 (Fig. 3). These results indicate Ser103 is the predominant ERF8 phosphorylation site by these MPKs. This finding was further validated by Liquid Chromatography-Mass Spectrometry (LC-MS/MS) phosphopeptide analysis as the most abundant ERF8 phosphopeptides identified following in vitro phosphorylation by MPK4 or MPK11 contain Ser103, with both 1 and 3 h kinase reactions (Table 1). In addition, mutating Ser103 to alanine or aspartic acid did not affect the in vitro phosphorylation of other potential phosphorylation residues of ERF8 (Additional file 4), indicating that Ser103 phosphorylation is likely not required for subsequent phosphorylation of additional residue(s).

**Table 1:** Mass spectrometry of MPK4 or MPK11 phosphorylated ERF8

mutation site	a.a.	MPK11/CAKK6 3 h rxn			MPK11/CAKK6 1 h rxn			MPK4/CAKK6 3 h rxn		
		total spectra	p-spectra	% p	total spectra	p-spectra	% p	total spectra	p-spectra	% p
		805 (89%)	41^a^	5.1	176 (84%)	10^b^	5.7	223 (84)%	30^c^	13.5
1	Thr69	102	0	–	22	0	–	31	1	13.5
	Ser93	141	3	2.1	31	1	3.2	46	0	3.2
	Ser97/98	143	1	0.7	32	1	3.1	47	6	–
2	Ser103	143	27	18.9	31	5	16.1	44	24	12.8
3	Thr111	112	9	9	14	0	–	11	0	54.5
	Ser118	112	1	0.9	14	0	–	11	0	–
	Thr142	50	0	–	20	0	–	19	1	5.3
	Thr147/148	85	1	1.2	33	1 (+ 2)	3	34	1	2.9+
	Ser153	99	0	–	7 (+ 31)	2	5.3	40	2	5
	Ser155	102	0	–	6 (+ 33)	0	–	43	1	2.3
4	Ser171	5	0	–	0 (+ 9)	0	–	0 (+ 19)	0	–

^a^One spectra was phosphorylated at two sites

^b^One spectra was phosphorylated at three sites

^c^Six spectra were phosphorylated at two sites

Summary of data obtained from LC-MS/MS analysis of GST-ERF8 protein after in vitro phosphorylation by GST-MPK4 or GST-MPK11. GST-ERF8 protein was excised following separation by SDS-PAGE and staining with Coomassie R-250. Numbers indicate the total number of peptide spectra observed for each site, as well as the total phosphorylated peptide spectra observed for those sites. Peptides identified with < 90% confidence were excluded from analysis, while (+n) indicates the presence of additional lower probability spectra in some cases. Total percentage sequence coverage of ERF8 is indicated in parentheses for each reaction

### Mutation of Ser103 alone is not sufficient to Alter ERF8-induced cell death

**Fig. 4:**
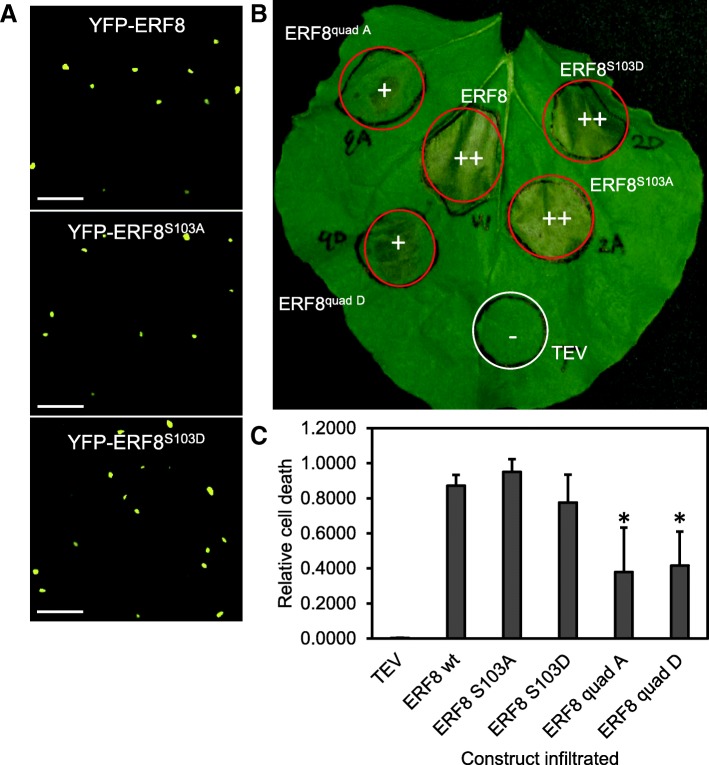
Mutation of all four phosphorylation sites weakens *ERF8*-induced cell death. (**a**) The nuclear localization of YFP-tagged ERF8 is not altered in the S103A and S103A mutants. Shown is transient expression of ERF8 in *N. benthamiana* 1 dpi. Scale bar = 50 μm. (**b**) Cell death triggered by ERF8 (W), ERF8^S103A^, ERF8^S103D^, ERF8^quad A^ and ERF8^quad D^ at 4 dpi. ++: strong cell death, +: weak/delayed cell death, −: no cell death. (**c**) Photos of leaves infiltrated with ERF8 variants at 4 dpi were analyzed for cell death severity. The fraction of cell death to healthy leaf tissue from each image was quantified using the ImageJ macro disease image-based quantification (PIDIQ) assay (LaFlamme et al., 2016). Shown are the mean ± SD (*n* = 3). Asterisks indicate statistical significance from ERF8 wt (student’s t test; *p* < 0.01)

To investigate the effect of ERF8 phosphorylation on cell death induction, the phosphomimetic (ERF8^S103D^) and phospho-dead (ERF8^S103A^) variants were transiently expressed in *N. benthamiana*. Neither mutation affected the localization of ERF8 to the nucleus (Fig. 4a), while expression of ERF8^S103D^ or ERF8^S103A^ each induced cell death in a manner similar to wildtype ERF8 (Fig. 4b, c). However, when all 4 phosphorylation residues, Ser93, Ser103, Thr111 and Ser171 were mutated to either alanine or aspartic acid (ERF8^quad A^, ERF8^quad D^), cell death was delayed and weaker than that induced by wildtype ERF8 (Fig. 4b, c). Western Blot indicated that both quadruple mutant protein levels were lower than wildtype (Additional file 5A), while RT-PCR analysis revealed that the mRNA levels of the wildtype and mutant versions of *ERF8* were comparable (Additional file 5B), suggesting that protein stability is affected in the quadruple mutants.

It was previously reported that flg22, a PAMP derived from bacterial flagellin, triggers MPK4-dependent phosphorylation of the EAR-motif possessing transcriptional repressor ARABIDOPSIS SH4-RELATED 3 (ASR3), which fine tunes defense-related transcriptional responses [61]. Since MPK4 and MPK11 are activated upon perception of flg22, we tested the phosphorylation status of ERF8 after flg22 treatment. *ERF8* expressed from Arabidopsis protoplasts following flg22 or ABA treatment were examined for a mobility shift, indicative of phosphorylation. As shown in Additional file 6 we did not observe a visible mobility shift in the molecular weight of ERF8 after flg22 or ABA treatment. This observation may be due to the low number of putative MPK-phosphorylated sites on ERF8, which are likely insufficient to induce a detectable change in electrophoretic mobility.

### ERF8 overexpression leads to differential expression of genes related to ABA signaling, cell death regulation and immunity

Our data suggest that transcriptional suppression of target genes by ERF8 is linked to its ability to induce PCD. To examine the downstream targets and signal transduction of ERF8, genome wide transcriptome analysis was performed by RNA-Seq using DEX-inducible *ERF8-*OE transgenic Arabidopsis plants. Following 8 h of DEX treatment, plant tissue was harvested for RNA extraction and processed for next generation sequencing. A large number of genes exhibited differential expression in the DEX treated *ERF8*-OE compared to the empty vector control samples. Eleven thousand seven hundred twenty-one genes showed a minimal of 2 fold change in its expression with 5886 genes that were up-regulated and 5835 genes that were down-regulated (Additional file 7). Genes upregulated by *ERF8* over-expression were enriched in biological processes (Gene Ontology (GO) terms) relevant for plant immunity, including systemic acquired resistance and SA-mediated signaling pathways, cell death and HR-related, defense response. On the other hand, GO terms for down-regulated genes included metabolic pathways, photosynthesis, and glucosinolate biosynthesis (Additional file 8).

As expected for ERF8 being a repressor of ABA responses, the ABA marker *RD29A* and a number of direct ABA target genes were down-regulated (Table 2). Additionally, of 169 genes that are directly up-regulated by ABA [42], 61 were down-regulated after ERF8 over-expression (Additional file 9). These genes include the biosynthesis genes *ABA1* and *NCED3*, the PP2C AHG3, *SnRK3.14*, and the transcription factors *ABF3*, *RGL3, NAC18, NAC72 (RD26), MYB77, CIR1*, and *At-HB12,* all of which were connected to ABA-related processes [42]. However interestingly, some ABA-marker genes like *RESPONSIVE TO ABA 18* (*RAB18*), *RESPONSIVE TO DESICCATION29B* (*RD29B*) and *KIN1* were significantly up-regulated, as were the biosynthesis genes, *NINE-CIS-EPOXYCAROTENOID DIOXYGENASE5* (*NCED5*) and ABSCISIC *ALDEHYDE OXIDASE3* (*AAO3*). The ABA receptors *PYL6, PYL4* and PYL*1* as well as *SnRK3.15* were also upregulated (Table 2), while the expression of the strongly ABA-inducible PP2Cs *HIGHLY ABA-INDUCED PP2C GENE 1* (*HAI1*) and *ABA INSENSITIVE1* (*ABI1*) [12, 42] did not change. This type of discrepancy in ABA-related gene expression upon cell death induction has also been observed in the SA-related autoimmunity mutants, *cpr22* and *ssi4* [12, 15], corroborating a role of ERF8 in ABA-SA crosstalk.

**Table 2:** Selected differentially regulated genes in *ERF8-*OE transgenic Arabidopsis plants 8 h after DEX treatment

Process	Name	AGI	Fold Change	*p-value*
Salicylic acid signaling	*NPR1*	AT1G64280	+ 4.2	5.13E-038
	*PR1*	AT2G14610	+ 18.3	2.49E-009
	*PR5*	AT1G75040	+ 45.5	1.98E-161
	*ICS1/SID2*	AT1G74710	+ 873.7	1.34E-238
	*EDS1*	AT3G48090	+ 26.1	4.50E-165
	*PAD4*	AT3G52430	+ 105.8	3.16E-175
	*RIN4*	AT2G17660	+ 18.2	0.017913
MAP kinase signaling in defense	*MEKK1*	AT4G08500	+ 4.9	7.27E-040
	*MKK4*	AT1G51660	+ 17.4	1.13E-099
	*MKK5*	AT3G21220	+ 9.4	7.46E-081
	*MEK1/MKK1*	AT4G26070	+ 5	4.26E-035
	*MKK2*	AT4G29810	+ 6.2	3.07E-070
	*MPK4*	AT4G01370	+ 4.9	5.73E-049
	*MPK11*	AT1G01560	+ 214.5	1.98E-166
	*MPK3*	AT3G45640	+ 16.1	8.97E-069
	*MPK6*	AT2G43790	+ 3.5	8.14E-031
	*WRKY33*	AT2G38470	+ 23.1	5.04E-017
	*ERF104*	AT5G61600	+ 4.6	2.21E-006
Cell death-related genes	*Bax inhibitor 1*	AT5G47120	+ 13.7	2.12E-113
	*Metacaspase 2*	AT4G25110	+ 33.7	1.56E-100
	*Metacaspase 5*	AT1G79330	+ 66.8	2.79E-006
	*Metacaspase 6*	AT1G79320	+ 278.6	1.83E-021
	*Metacaspase 7*	AT1G79310	+ 11.2	7.43E-007
	*Metacaspase 8*	AT1G16420	+ 227.9	6.34E-128
	*BAG2*	AT5G62100	+ 10.2	5.25E-034
	*BAG6*	AT2G46240	+ 2.4	5.83E-013
	*ACD11*	AT2G34690	−3.0	9.60E-023
	*LSD1*	AT1G62830	−2.3	9.75E-008
	*DND1*	AT5G15410	−55.3	1.51E-155
	*DND2*	AT5G54250	−24.4	9.10E-103
ABA signaling: ABA marker genes	*RD29A*	AT5G52310	−5.2	1.78E-015
	*RD29B*	AT5G52300	+ 5.9	2.20E-007
	*RAB18*	AT1G43890	+ 3.3	1.26E-029
	*KIN1*	AT1G14370	+ 3.3	9.78E-026
ABA receptors	*PYL1*	AT5G46790	+ 3.2	2.85E-025
	*PYL4*	AT2G38310	+ 5.9	2.44E-025
	*PYL6*	AT2G40330	+ 41.5	3.58E-067
SnRK kinases	*SnRK3.15*	AT5G01820	+ 2.3	1.57E-013
	*SnRK3.14*	At4g30960	−7.4	7.44E-060
PP2Cs	*ABI1*	AT4g26080	NS	–
	*HAB1*	At1g72770	NS	–
	*HAI1*	AT5g59220	NS	–
	*AHG3*	AT3G11410	−4.3	2.06E-031
ABA biosynthesis	*ABA1*	At5g67030	−5.9	5.56E-024
	*AAO3*	AT2G27150	+ 3.0	5.34E-021
	*NCED3*	At3g14440	−3.6	3.90E-016
	*NCED5*	AT1G30100	+ 8.7	2.65E-015
Transcription factors	*ABF3*	AT4g27410	−17.3	3.18E-007
	*CIR1*	At5g37260	−19.7	5.69E-044
	*At-HB12*	At3g61890	−28.8	1.95E-018
	*RGL3*	AT5G17490	−4.7	1.00E-006
	*NAC72 (RD26)*	At4g27410	−3.0	3.18E-007
	*NAC18*	At1g52880	−2.6	3.62E-008
	*Myb77*	At3g50060	−2.2	0.007121
Ethylene signaling	*ACS7*	AT4G26200	+ 1297	2.73E-245
	*ACS2*	AT1G01480	+ 4.5	0.003495
	*ACS6*	AT4G11280	+ 5.8	1.48E-005
	*ERS1*	AT2G40940	+ 2.1	1.80E-012
	*EIN4*	AT3G04580	+ 6.6	1.14E-067
	*EIN3*	AT3G20770	NS	5.08E-012
	*ERF1*	AT3G23240	+ 369.5	2.37E-209
	*ERF4*	AT3G15210	−28.8	1.36E-028
	*ERF8*	AT1G53170	+ 127	2.07E-278
	*PDF1.2*	AT5G44420	NS	–
	*CHI-B*	AT3g12500	NS	–

Fold change is relative to DEX treated empty vector transgenic plants. NS = no significant change

Although ERF8-induced cell death was not dependent on SA biosynthesis (Fig. 2b), well characterized SA-mediated pathogen defense-related genes including *NON EXPRESSOR OF PATHOGENESIS-RELATED GENES 1* (*NPR1*), *PHYTOALEXIN DEFICIENT4* (*PAD4*), *ENHANCED DISEASE SUSCEPTIBILITY 1* (*EDS1*), *RPM1-INTERACTING4* (*RIN4*)*, PR1, PR5,* as well as the SA biosynthesis gene *SID2* were significantly induced (Table 2). The up-regulation of genes involved in SA biosynthesis and signal transduction could be attributed by the activation of the SA positive feedback loop [62, 63], in accordance with our hypothesis that ERF8 acts downstream of SA accumulation. Furthermore, pathogen defense-related MAP kinase cascade components were induced. These include *MPK3*, *4*, *6* and *11*, their upstream MKKs as well as their downstream targets *WRKY DNA BINDING PROTEIN 33 (WRKY33), ERF104, ACC SYNTHASE 2 (ACS2)* and *ACS6* that mediate transcriptional changes of defense-related genes upon pathogen invasion [64–67]. Interestingly, *MPK11* was up-regulated more than 200-fold. In contrast to the upregulation of SA signaling, the majority of ethylene-associated genes including the marker genes *PDF1.2* and *CHI-B* did not show significant transcriptional changes even though the ethylene biosynthesis genes *ACC synthase* (*ACS*) *7*, *ACS6* and *ACS2* were induced (Table 2). Several class IX *ERF* genes, *ERF1*, *ERF2,* and *ERF6,* which have been implicated in immunity [29–32], were also up-regulated, while most other class VIII EAR repressor *ERFs* showed no significant expression changes. However, *ERF8*’s closest paralog, *ERF4,* was strongly down-regulated (29 fold), indicating transcriptional feedback regulation. JA-related marker genes displayed either no change (*Myc2*, *LOX2*) or were down-regulated (*VSP2*, *AOS*). This could be due to the up-regulation of SA signaling at the 8 h time point.

As expected, many cell death-related genes were differentially expressed. These include genes encoding for positive and negative regulators of cell death, as well as genes that elicit PCD when they are mis-regulated. For example, the expression of both type I and type II metacaspases (*MC2–8*) [68], which are thought to be positive regulators of PCD [69], increased by 2.2- to 378-fold (Table 2). Furthermore, a group of genes that negatively regulate HR-like PCD, including *ACCELERATED CELL DEATH11* (*ACD11*), *LESION STIMULATING DISEASE RESISTANCE RESPONSE1* (*LSD1*), *DEFENSE, NO DEATH1* and *2* (*DND1*, *DND2*) [70–72], were down regulated (Table 2). Taken together, the transcriptomic changes associated with *ERF8* supports its role as a positive regulator of PCD and suggests its potential role in pathogen defense.

### ERF8 positively regulates bacterial immunity

**Fig. 5:**
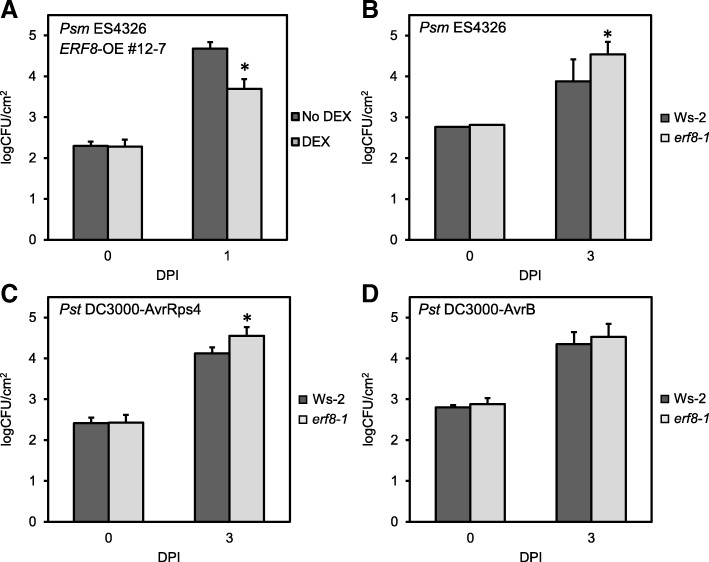
*ERF8* is involved in defense against *Pseudomonas syringae.* Virulent *P.s*. pv maculicola ES4326 (*Psm*) was pressure infiltrated at an OD_600_ of 0.0001 into control or DEX-treated *ERF8-OE* plants (**a**) or *erf8–1* knockdown plants (**b**). Avirulent *Pseudomonas syringae* pv. *tomato* DC3000 (Pst) carrying AvrRps4 (**c**) or AvrB (**d**) was pressure infiltrated into wildtype Ws-2 and *erf8–1* knockdown plants. *In planta* bacterial growth was quantified at 1 or 3 days post inoculation (dpi). Asterisks indicate statistical significance (student’s t test; *p* < 0.01). Experiments were conducted twice (C&D) or 3 times (A&B) and showed reproducible results (n = 3 for 0 dpi, *n* = 8–10 for 1 or 3 dpi)

Given the results of our cell death assays and genome-wide transcriptional analyses, we investigated whether ERF8 plays a role in plant immunity. *ERF8-OE* or *erf8–1* plants were infected with various strains of the hemi-biotrophic bacterial pathogen, *P. syringae*. As shown in Fig. 5, DEX-treated *ERF8-OE* Arabidopsis lines displayed increased resistance against the virulent strain, *P. syringae* pv*. maculicola* ES4326 *(Psm* ES4326) compared to control plants, suggesting a positive role of ERF8 in pathogen resistance (Fig. 5a). Supporting this observation, *erf8–1* plants showed enhanced susceptibility compared to the corresponding wildtype, Ws-2 against *Psm* ES4326 (Fig. 5b). Interestingly, *erf8–1* plants showed more growth of avirulent *P. syringae* pv*. tomato* (*Pst)* DC3000 carrying AvrRps4 (Fig. 5c) whereas no alteration in pathogen growth was observed with *Pst* DC3000 carrying AvrB (Fig. 5d), indicating a role of ERF8 in immunity.

## Discussion

In this study, we investigated a class II ERF transcriptional repressor, ERF8, which induces PCD in two model plant species, *A. thaliana* and *N. benthamiana*. Mutation of the conserved transcriptional suppression EAR motif completely abolished the ability of *ERF8* to induce cell death, indicating its transcriptional repression activity is essential for cell death induction. These data corroborate previous studies of proposed roles for class II ERFs in cell death induction [36, 52, 53].

Previous studies have revealed different modes of regulation of ERF transcription factors, including co-transcriptional regulators, miRNA, alternative polyadenylation, phosphorylation and proteasome-mediated degradation [28, 30, 31, 36, 73]. ERF8 contains a putative MAP kinase docking site, and in this study we demonstrated two immunity-related kinases, MPK4 and MPK11, interact with and directly phosphorylate ERF8. MPK4 in particular has been studied extensively in the context of plant immunity [43–47]. The *mpk4* mutant exhibits extreme dwarf phenotypes and autoimmunity including spontaneous PCD [45, 74]. Several MPK4 interactors and/or substrates have been previously identified, including MAP KINASE SUBSTRATE 1 (MKS1), which is required the SA-dependent autoimmunity phenotype of *mpk4* [75]. MKS1 phosphorylation by MPK4 occurs following pathogen infection, leading to WRKY33-induced expression of PAD3, which is required for biosynthesis of the antimicrobial phytoalexin, camalexin [65]. Another target of MPK4 is the PROTEIN ASSOCIATED WITH TOPOISOMERASE II (PAT1), a component of the mRNA decapping machinery [76]. PAT1 also interacts with the CC-NB-LRR protein, SUMM2 (SUPPRESSOR OF mkk1 mkk2), mutation of which can partially suppress autoimmune phenotypes of *mpk4* [76, 77]. The absence of PAT1 triggers SUMM2-dependent immunity, and based on these findings, it has been proposed that *MPK4* positively regulates PTI and also acts as a guardee, suggesting that the loss of MPK4 induces SUMM2-mediated ETI [76, 77]. Another MPK4 interactor is the transcriptional repressor ARABIDOPSIS SH4-RELATED3 (ASR3), which negatively regulates a large subset of flg22-induced genes. Phosphorylation of ASR3 by MPK4 enhances its DNA binding activity to suppress gene expression [61]. It will be interesting to determine if ERF8 interacts with some of these known targets of MPK4 and functions within the same protein complexes or in an overlapping signal transduction pathway leading to pathogen resistance and PCD induction.

Given the roles of *MPK4* and *MPK11* in immunity, we hypothesized that *ERF8*-induced cell death is regulated by phosphorylation. Our in vitro kinase assays and LC-MS/MS results indicate MPK4 and MPK11 predominantly phosphorylate ERF8 at residue Ser103. Thus, phosphomimetic and phosphoablative variants in this single residue (ERF8^S103D/A^) were generated and overexpressed in *N. benthamiana*, but no significant difference in cell death was observed relative to wildtype ERF8. However, in contrast, when all 4 potential phosphorylation residues Ser93, Ser103, Thr111 and Ser171 were mutated to alanine or aspartic acid, ERF8-induced cell death was reproducibly delayed and weakened. It is likely that the phosphomimetic mutations failed to reproduce changes to ERF8 caused by phosphorylation due to differences in charge and size between an aspartate residue and a phosphate group [78], which suggests phosphorylation by MPK4 an MPK11 positively regulates ERF8 to induce cell death. The sequence coverage in our LC-MS/MS data is low for the 4th potential phosphorylation residue Ser171 (Table 1), presumably due to its proximity to the C-terminal end of the protein, and thus it remains possible that other site(s) along with Ser103 are phosphorylated by MPK4 and MPK11 in vivo to regulate cell death. Phosphorylation may stabilize the ERF8 protein level, as the quadruple phosphosite mutants displayed lower protein accumulation *in planta*, while mRNA levels were unaffected (Additional file 5). The induction or activation of *MPK11* and/or *MPK4* upon pathogen infection may therefore reduce ERF8 turnover and contribute to PCD formation. Regulation of ERF8 through turnover by the 26S proteasome was previously suggested [36]; it was shown that treatment with the proteasome inhibitor MG132 slowed down protein turnover. Protein turnover of ERF8 (and ERF4) was also slowed down as plants aged and it was suggested that this would contribute to the induction or execution of senescence-associated cell death [36]. Further analysis of the mechanism(s) of ERF8 phospho-regulation is on-going.

As previously shown for other class VII ERFs, including *ERF7* and *ERF4, ERF8* acts as a negative regulator of ABA responses [39, 42, 54] (Fig. 1). It is believed that ERFs bind to the GCC and DRE elements in promoters and form a repressor complex with co-repressors, such as TOPLESS, histone deacetylase HDA19 and its interactor Sin3 [39, 79].

In this study, ERF8 over-expression led to the down-regulation of 5835 genes. Many of those are probably altered because of secondary effects due to elevated SA levels, but the fact that 36% of genes that are directly induced by ABA [42] were down-regulated after induction of *ERF8* expression strongly suggests that ERF8 is indeed a transcriptional repressor of ABA responses. Interestingly, one of the strongly down-regulated genes was *ERF4*, the closest paralog of *ERF8*, indicating cross-regulation between these transcription factors. It had been suggested that both ERF4 and ERF8 co-regulate senescence through their repressor function and over-expression of either gene caused reduced ABA sensitivity [36, 54] (Fig. 1), however, Caarls et al. (2017) [80] showed that ERF8 but not ERF4 regulates *PDF1.2* and *PR1* gene expression, suggesting distinct roles for these two transcription factors. ERF8 was recently also identified as part of a network of ERF transcription factors (together with ERF6, ERF9, ERF59 and ERF98) that controls osmotic stress [81].

Olvera-Carrillo et al. (2015) [82] previously identified transcriptional profiles differentiating HR-like (biotic stress) cell death from developmental cell death. The ERF8 set overlapped in 27 out of 28 genes that were up-regulated in their biotic stress set, while only 8 of 25 developmental PCD marker genes were up-regulated, 11 did not change and 6 were down-regulated, confirming the immunity-specific nature of ERF8-induced cell death. However, transcriptional changes at our selected time point (8 h post *ERF8* induction) seemed sufficient to trigger extensive secondary transcriptional reprogramming in Arabidopsis. Particularly, the SA biosynthesis gene *ICS1* (*SID2*) was upregulated strongly and thus it likely led to SA-mediated secondary transcriptional changes (a positive amplification loop), making it difficult to identify direct ERF8 target genes. Three hundred genes with a GCC box in their promoter (− 2000 to + 200; [83]) were down-regulated and 249 were up-regulated. Similarly more than 200 genes with a DRE element in their promoter were up or down regulated, respectively (Additional file 8). Interestingly, of the 61 genes that are directly up-regulated by ABA but down-regulated in our data set (Additional file 9) 33 (54%) contained a DRE element in their promoter, 3 had a GCC element and 4 contained both GCC and DRE elements. Furthermore, the down-regulated ABA-related genes *RD29A*, *RD26/NAC072* also contain a DRE element, *SnRK3.14* a GCC element, and *NCED3* and *HB12* GCC and DRE elements in their promoters. These could be direct ABA-related targets of ERF8. The down-regulated cell death-related genes, *DND1* and *ACD11,* also contain DRE elements in their promoters. *DND1* has been shown to be a target of Topless-related 1 (TPR1) [72] and topless proteins have been shown to interact with ERF8 [79] raising the possibility that ERF8 and topless proteins may be co-suppressors of *DND1* and potentially other genes from our data set. Further analyses to identify the immediate target(s) of ERF8 will be the key to understanding how this transcriptional repressor integrates ABA and cell death.

Finally, we demonstrated the functional role of ERF8 in immunity, as overexpression in Arabidopsis increased resistance against *Psm* ES4326, while *erf8–1* plants exhibited enhanced susceptibility to virulent *Psm* ES4326 as well as *Pst* DC3000 expressing AvrRps4. Cumulatively, our data demonstrate that ERF8 functions in both ABA signaling and bacterial immunity. The attenuation of resistance to *Pst* AvrRps4 suggests a link to ETI conferred by TIR-NB-LRR class *R*-genes like *SNC1* [72]. Indeed, in a separate study (Cao et al., submitted) we show that ERF8 is targeted by multiple *P. syringae* type III effector proteins, further corroborating an important role of ERF8 in plant immunity. Further study of the ERF8-mediated crosstalk with ABA signaling will be a promising avenue to understand the transcriptional network in abiotic and biotic responses.

## Conclusions

In this study, we revealed that the ABA-inducible transcriptional repressor ERF8 has dual roles in ABA signaling and immunity. ERF8 acts as a negative regulator of ABA signaling as the *erf8* knockdown line displayed enhanced ABA sensitivity while overexpression lines showed decreased sensitivity. Additionally, over-expression of ERF8 caused SA-independent PCD as well as enhanced pathogen resistance, suggesting a positive role in plant immunity. However, the EAR repressor domain was required for PCD formation and a number of PCD-associated genes were down-regulated in ERF8 overexpression lines, suggesting that ERF8 may down-regulate negative regulators of immunity signaling. Finally, we show that ERF8 is phosphorylated by the two immunity-related MAP kinases, MPK4 and MPK11. Ser103 was predominantly phosphorylated in vitro; however mutation of all four putative phosphorylation sites seemed to be necessary to partially suppress *ERF8*-induced cell death in *N. benthamiana.*

## Methods

### Plant growth conditions

*Arabidopsis thaliana* and *Nicotiana benthamiana* seeds were grown in Sunshine Mix (Sun Gro Horticulture Canada) in a growth chamber at 9 h light and 16 h dark cycles at 22 °C and 20 °C respectively. Light intensity was approximately 130 μE m^− 2^ s^− 1^.

### Generation of Arabidopsis transgenic plants

Arabidopsis plants were transformed following the floral dip method using *Agrobacterium* (strain GV3101) carrying the binary plant expression vector pMAC14 containing a DEX-inducible *ERF8-HA-tag* construct. BASTA-resistant individuals were selected and leaves from 5-week-old T1 transformants were treated with 30 μM DEX for 2 days, after which leave samples were frozen in liquid nitrogen and prepared for western blotting to detect protein expression of the transgene. T1 individuals with confirmed transgene expression were carried to the next generation and homozygous T3 transgenic lines were used for experiments. The *ERF8* knockdown line FLAG157D10 was obtained from the ABRC stock center.

### Germination assays

Sterilized seeds were plated onto 0.5X Murashige and Skoog (MS) media (pH 5.8; Sigma) supplemented with ABA (Sigma) or dexamethasone (DEX; Bioshop). Germination was scored based on radicle or cotyledon emergence observed under a dissecting microscope.

### Cloning and agrobacterium-mediated transient expression in N. Benthamiana

Wildtype and mutant variants of *ERF8*, *MPK4*, and *MPK11* were sub-cloned using gateway LR clonase II (Invitrogen) into the binary expression vectors pBWGYn2 or pBWGYc2 (for bimolecular fluorescence complementation, BiFC), pEARLEY201 (for protein expression with a C-terminal HA tag), or pEARLEY104 (with an N-terminal YFP tag for cellular localization analyses). *A. tumefaciens* (GV2260 or C58C1) were transformed with the constructs and transient assays were conducted as described [84]. *Agrobacterium* carrying CaMV35S::HC-Pro from *tobacco etch virus* (TEV) was co-infiltrated with constructs to suppress gene silencing. For BiFC and co-infiltration experiments, equal volumes of cultures were mixed prior to infiltration. Six week old *N. benthamiana* leaves were infiltrated from the underside using needleless syringes as described previously [84].

### Confocal microscopy

Discs were cored from infiltrated *N. benthamiana* leaf areas and imaged using the Leica TCS SP5 confocal system (Leica Microsystems). Images were acquired with the Argon laser set to 20%, using excitation at 514 nm and emission from 525 to 600 nm for YFP detection. Autofluorescence of chloroplasts was detected by emission between 650 and 700 nm [36].

### Western blotting

Arabidopsis and *N. benthamiana* tissue was frozen in liquid nitrogen and ground into fine powder. Proteins were extracted in 20 mM TRIS-HCl pH 8.0, 100 mM NaCl, 1 mM DTT and 1.25% Triton X-100. Samples were centrifuged at 6000×*g* for 10 min at 4 °C to remove debris. Resulting protein extracts were boiled in 1X SDS loading dye at 90 °C for 5 min. After Western blotting proteins were detected using α-HA antibodies (1:10,000; Roche) and peroxidase-conjugated mouse α -rabbit IgG (1:30,000; Cell Signaling). Immuno-reactive bands were detected using the ECL prime western blotting kit (GE Healthcare).

### In vitro kinase assays

ERF8, MPK, and CA-MKK proteins used in kinase assays were expressed as recombinant GST-fusion proteins in *E. coli* BL21 codon plus cells using the pGEX4T3 expression vector. GST-fusion proteins were purified via GSH-agarose chromatography and eluted in 50 mM HEPES, 100 mM NaCl, 5% glycerol, 1 mM DTT, pH 7.5 by addition of 10 mM reduced GSH. Proteins were quantified and stored at − 80 °C until use. For kinase assays, 1 μg of GST-ERF8 (WT or mutant as indicated in figures) was incubated with 0.5 μg GST-CA-MKK and 0.5 μg GST-MPK in 25 mM Tris-Cl, 10 mM MgCl_2_, 1 mM Na_3_VO_4_, 1 mM β-glycerophosphate, pH 7.5. Reactions were initiated by addition of ATP (final concentration 100 μM ATP + 2 μCi [γ-^32^P]-ATP). Following incubation at 30 °C for 2 h reactions were stopped by addition of 1X SDS loading dye and heating to 95 °C for 10 min. Proteins were separated on 15% SDS-PAGE and transferred to nitrocellulose. Radiolabeled proteins were detected by film exposure for times indicated in figures.

### LC-MS/MS

In vitro kinase assays were performed as described above with the following changes. Assays were conducted with unlabeled ATP, and used 5 μg GST-ERF8 protein and 1 μg each GST-CA-MKK6 and GST-MPK4 or GST-MPK11 (as indicated in figures). Reactions were incubated for 1 or 3 h as described in figures. GST-ERF8 protein was excised following separation by SDS-PAGE and staining with Coomassie R-250 and sent for in-gel trypsin digestion and LC-MS/MS phosphopeptide analysis (Mass Spectrometry Facility, SPARC BioCentre).

### Visualization of microscopic cell death by trypan blue staining

Leaves were submerged in lactophenol-trypan blue solution, heated in boiling water in a fume hood for 3–5 min and stained at room temperature for 2 h. Samples were destained in 50% chloral hydrate (*w*/*v*) overnight, washed and stored in 50% glycerol before mounting onto glass slides for images analysis and photography.

### Quantification of cell death

Images were pre-processed using Photoshop. Individual infiltration spots were cropped and saved as separate images. Area outside the infiltrated tissue was filled in red to allow infiltrated leave tissue to be distinguished from un-infiltrated leave tissue. The fraction of cell death to healthy leaf tissue from each image was quantified using the ImageJ macro disease image-based quantification (PIDIQ) [85] with the following change in parameters: green area (hue: 50–104; saturation: 151–255; brightness: 0–255); cell death area (hue: 0–255; saturation: 0–150; brightness: 0–255). The average fraction of cell death from at least 3 leaves was depicted in figures.

### Protoplast transfection assay

Protoplast transfection assay was performed as previously described (Li et al., 2015). Briefly, 200 μl of Arabidopsis protoplasts at 2 × 10^5^ cells/ml were transfected with 40 μg plasmids expressing ERF8 or its variants or MKP phosphatase. Twelve hours after transfection, the protoplasts were treated with 100 nM flg22 for 15 min or 10 μM ABA for 15 or 30 min. For K252a treatment, 1 μM K252a was added to the protoplasts right after transfection. The protoplasts were isolated for Western Blot analysis with anti-HA antibody.

### RNA extraction and Illumina mRNA-Seq methods

Three replicates were used for each genotype (empty vector and *ERF8*-OE 8 h after DEX treatment). Each replicate contains 3 leaves pooled from 3 plants that were 4 weeks old. RNA was extracted from plant tissues using the TRIzol reagent following the manufacturer’s protocol (Thermo Fisher Scientific, Waltham, MA USA). The RNA was further purified using the PureLink™ RNA Mini Kit (Thermo Fisher Scientific, Waltham, MA USA). RNA was bound, washed and eluted following the manufacturer’s protocol. Eluted RNAs were treated with the Turbo DNA-*free*™ kit (Thermo Fisher Scientific, Waltham, MA USA) to remove residual double-stranded DNA following the protocol from the manufacturer. The quality and quantity of the RNAs were assessed using the RNA Nano kit for the Bioanalyzer 2100 (Agilent Technologies, Palo Alto, CA). mRNA was isolated from the total RNA using the Dynabeads® mRNA Purification Kit (Thermo Fisher Scientific, Waltham, MA USA) following the recommended protocol. The mRNA was sheared to 300 base size using the Covaris S2 Ultrasonicator (Covaris, Woburn, MA) with the following protocol: duty = 10%; intensity = 5%; cycles per burst = 200 and time = 35 s X 2. The sheared mRNA was precipitated overnight and resuspended in 14ul of RNase-free water.

Illumina libraries were prepared using the NEBNext® mRNA Library Prep Master Mix set for Illumina kit (New England Biolabs, Ipswich, MA, USA) starting at the first strand cDNA synthesis step and following the recommended protocol. NEB single index barcodes were added for multiplexing purposes. The final library was sequenced on the NextSeq500 sequencer (Illumina, San Diega, CA), according to the manufacturer’s instructions, using the 150 cycle Mid Output V2 sequencing kit and generating 150X2 paired end reads.

### RNA-Seq data processing

RNA-Seq reads were mapped to Arabidopsis gene sequences with the short read mapper novoalign (novocraft.com). The homopolymers and reads with low qualities were filtered by the mapper. The number of reads mapped to each gene were subsequently counted for each sample. The read count data were inputted to the R package edgeR [86] for gene differential expression analysis. Differentially expressed genes were grouped into up-regulated genes and down-regulated genes.

Sequence read numbers ranged from 8.2 to 17.6 million for each ERF8-OE or empty vector (EV) control sample, of which more than 83% were uniquely mapped to Arabidopsis genes, while only 0.4% of the reads were mapped to multiple locations. About 0.6% of the reads were filtered by the aligner due to being homopolymers. Around 14% of the reads have no match reported, presumably due to low quality. The reproducibility of the samples was assessed with the coefficient of determination for the gene-wise read counts data, which ranged 96–98% within the same genotype and 36–46% between different genotypes. Differentially expressed genes were searched for 1) GCC box/motif (TAAGAGCCGCC or AGCCGCC) in promoter region -2000 bp to + 200 bp and/or 2) DRE motif (A/GCCGAC) in promoter region -2000 bp to 0 bp.

### P. Syringae growth assays

*P. syringae* strains were inoculated at OD_600_ 0.002 for *Pma* ES4326, OD_600_ 0.001 for *Pst* DC3000, or OD_600_ 0.002 for *Pst* DC3000-AvrRps4 and *Pst* DC3000-AvrB. To quantify *in planta* bacterial growth, four disks (1 cm^2^) per plant were harvested, ground in 10 mM MgCl_2_, and plated on King’s broth (KB) with the appropriate antibiotics for colony counting. DEX-inducible transgenic plants were sprayed with 30 μM DEX 1 day in advance to induce transgene expression.

## Additional files

Additional file 1: **Figure S1.** ERF8 expression in seeds and after DEX treatment. (PPTX 512 kb)

Additional file 2: **Figure S2.** Localization and expression of ERF8 wildtype and L176A L178A mutant variant in *N. benthamiana*. (PPTX 6667 kb)

Additional file 3: **Figure S3.** Interaction of ERF8 and MPK11 in Y2H. (PPTX 1630 kb)

Additional file 4: **Figure S4.** Phosphorylation of Ser103 does not affect phosphorylation of other ERF8 residues in vitro. (PPTX 204 kb)

Additional file 5: Transient expression of *ERF8* wt and variants in *N. benthamiana*. (PPTX 129 kb)

Additional file 6: **Figure S6.** Analysis of ERF8 and its phosphorylation status under various conditions. (PPTX 154 kb)

Additional file 7: **Table S7.** Differentially regulated genes in *ERF8*-OX plants 8 h after DEX treatment. (XLSX 789 kb)

Additional file 8: **Figure S8.** Analysis of differentially expressed genes. (PPTX 39 kb)

Additional file 9: **Table S9.** Genes up-regulated by ABA and down-regulated in ERF8-OX plants. (XLSX 11 kb)

## Abbreviations

ABA: abscisic acid
BiFC: bimolecular fluorescence complementation
DEX: dexamethasone
EAR: ERF-associated amphiphilic repression (EAR)
HR: hypersensitive response
OE: over-expresser
PCD: programmed cell death
*Pst*: *P. syringae* pv*. tomato*
SA: salicylic acid
