# Supplementary material for: Arabidopsis ETHYLENE RESPONSE FACTOR 8 (ERF8) has dual functions in ABA signaling and immunity
Source: BMC Plant Biol. 2018 Sep 27;18:211. doi: 10.1186/s12870-018-1402-6 (PMC6161326; doi:10.1186/s12870-018-1402-6)
Supplement: Supplementary file 1 — Figure S1. ERF8 expression in seeds and after DEX treatment. (PPTX 512 kb) [file 12870_2018_1402_MOESM1_ESM.pptx]

## Slide 1
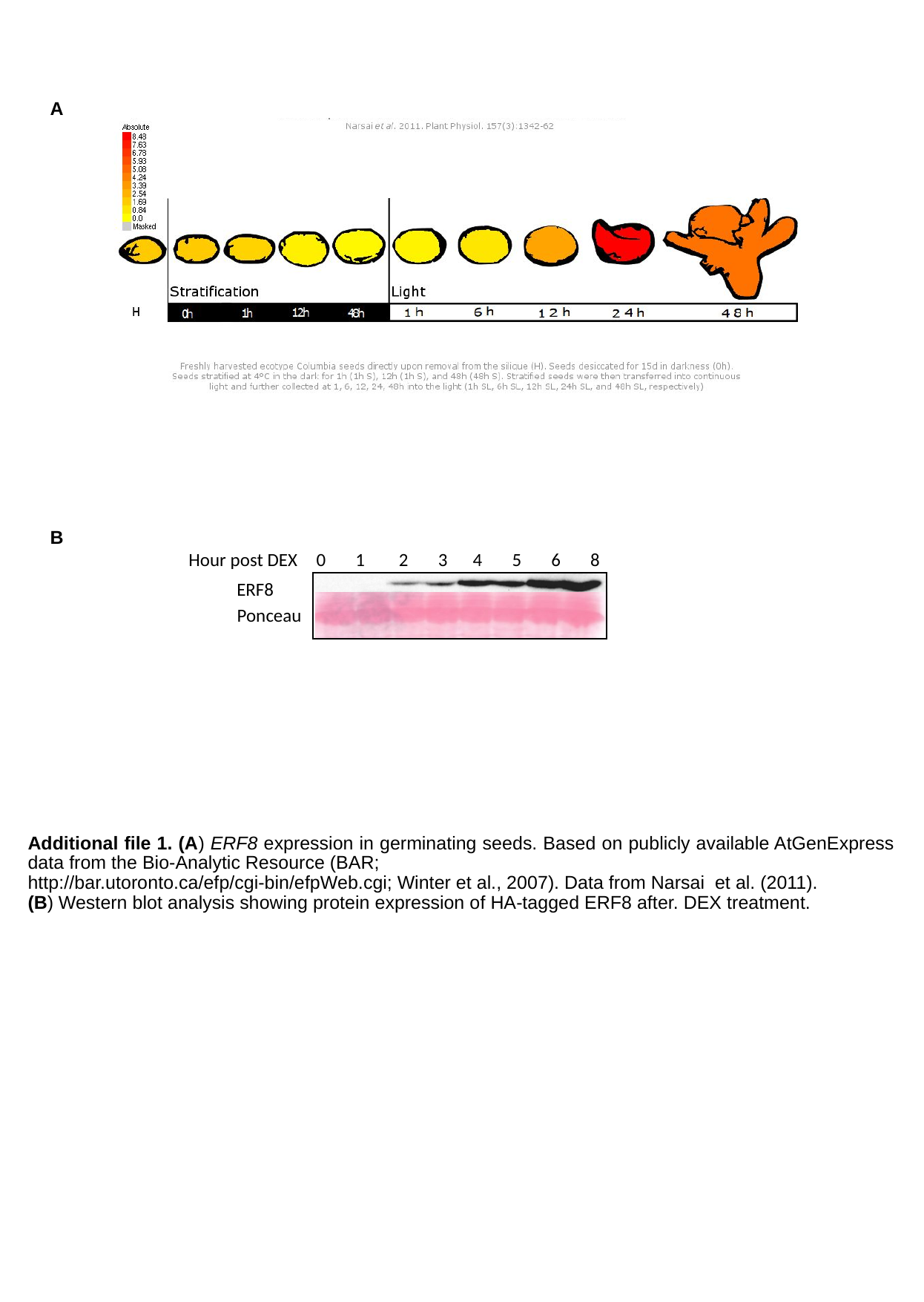

A
B
Hour post DEX
 0 1 2 3 4 5 6 8
ERF8
Ponceau
Additional file 1. (A) ERF8 expression in germinating seeds. Based on publicly available AtGenExpress data from the Bio-Analytic Resource (BAR;
http://bar.utoronto.ca/efp/cgi-bin/efpWeb.cgi; Winter et al., 2007). Data from Narsai et al. (2011).
(B) Western blot analysis showing protein expression of HA-tagged ERF8 after. DEX treatment.
