# Supplementary material for: Arabidopsis ETHYLENE RESPONSE FACTOR 8 (ERF8) has dual functions in ABA signaling and immunity
Source: BMC Plant Biol. 2018 Sep 27;18:211. doi: 10.1186/s12870-018-1402-6 (PMC6161326; doi:10.1186/s12870-018-1402-6)
Supplement: Supplementary file 2 — Figure S2. Localization and expression of ERF8 wildtype and L176A L178A mutant variant in N. benthamiana. (PPTX 6667 kb) [file 12870_2018_1402_MOESM2_ESM.pptx]

## Slide 1
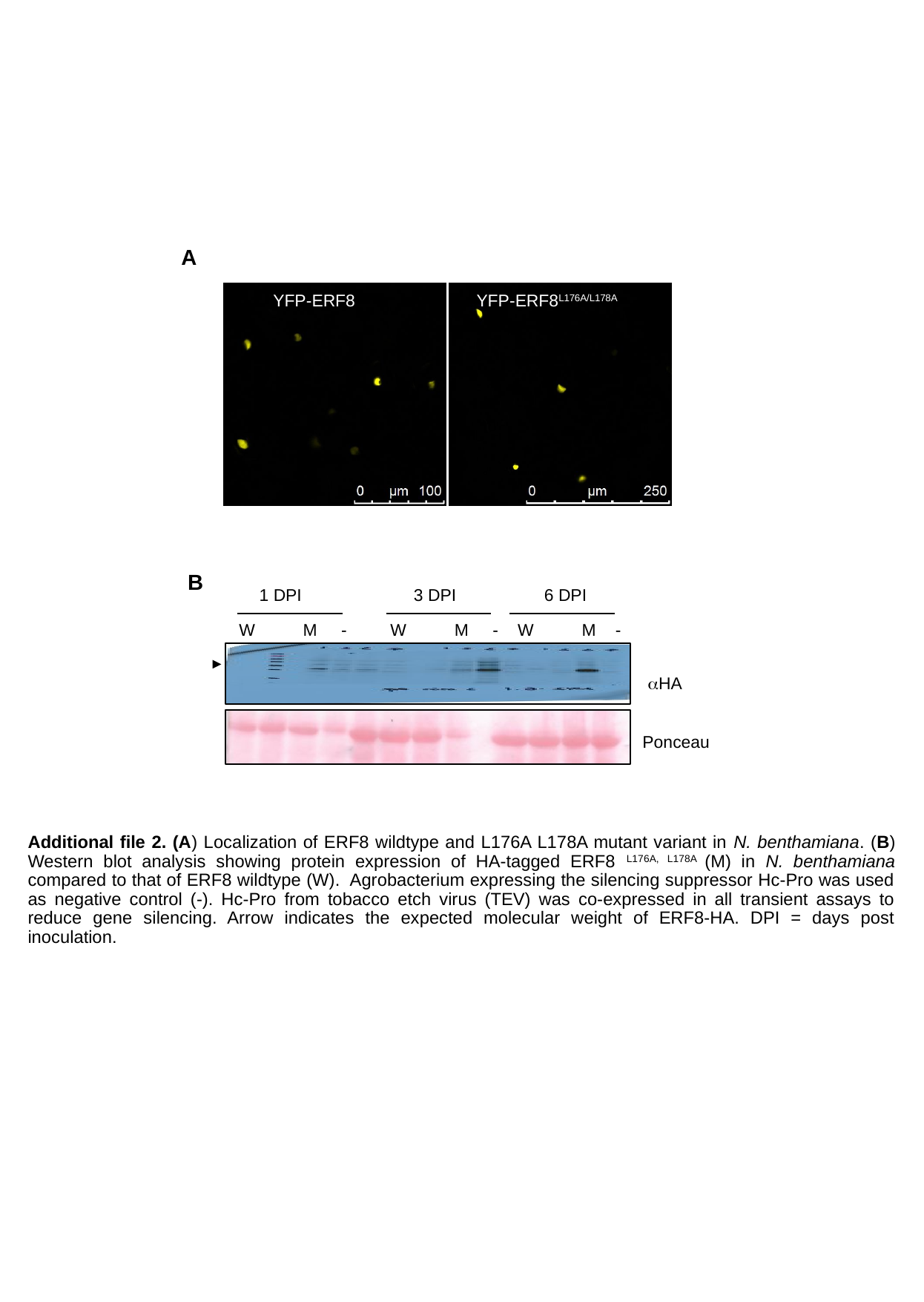

A
YFP-ERF8
YFP-ERF8L176A/L178A
B
 1 DPI 	 3 DPI	 6 DPI
 W M - W M - W M -
αHA
Ponceau
# Additional file 2. (A) Localization of ERF8 wildtype and L176A L178A mutant variant in N. benthamiana. (B) Western blot analysis showing protein expression of HA-tagged ERF8 L176A, L178A (M) in N. benthamiana compared to that of ERF8 wildtype (W). Agrobacterium expressing the silencing suppressor Hc-Pro was used as negative control (-). Hc-Pro from tobacco etch virus (TEV) was co-expressed in all transient assays to reduce gene silencing. Arrow indicates the expected molecular weight of ERF8-HA. DPI = days post inoculation.
