# Supplementary material for: Arabidopsis ETHYLENE RESPONSE FACTOR 8 (ERF8) has dual functions in ABA signaling and immunity
Source: BMC Plant Biol. 2018 Sep 27;18:211. doi: 10.1186/s12870-018-1402-6 (PMC6161326; doi:10.1186/s12870-018-1402-6)
Supplement: Supplementary file 3 — Figure S3. Interaction of ERF8 and MPK11 in Y2H. (PPTX 1630 kb) [file 12870_2018_1402_MOESM3_ESM.pptx]

## Slide 1
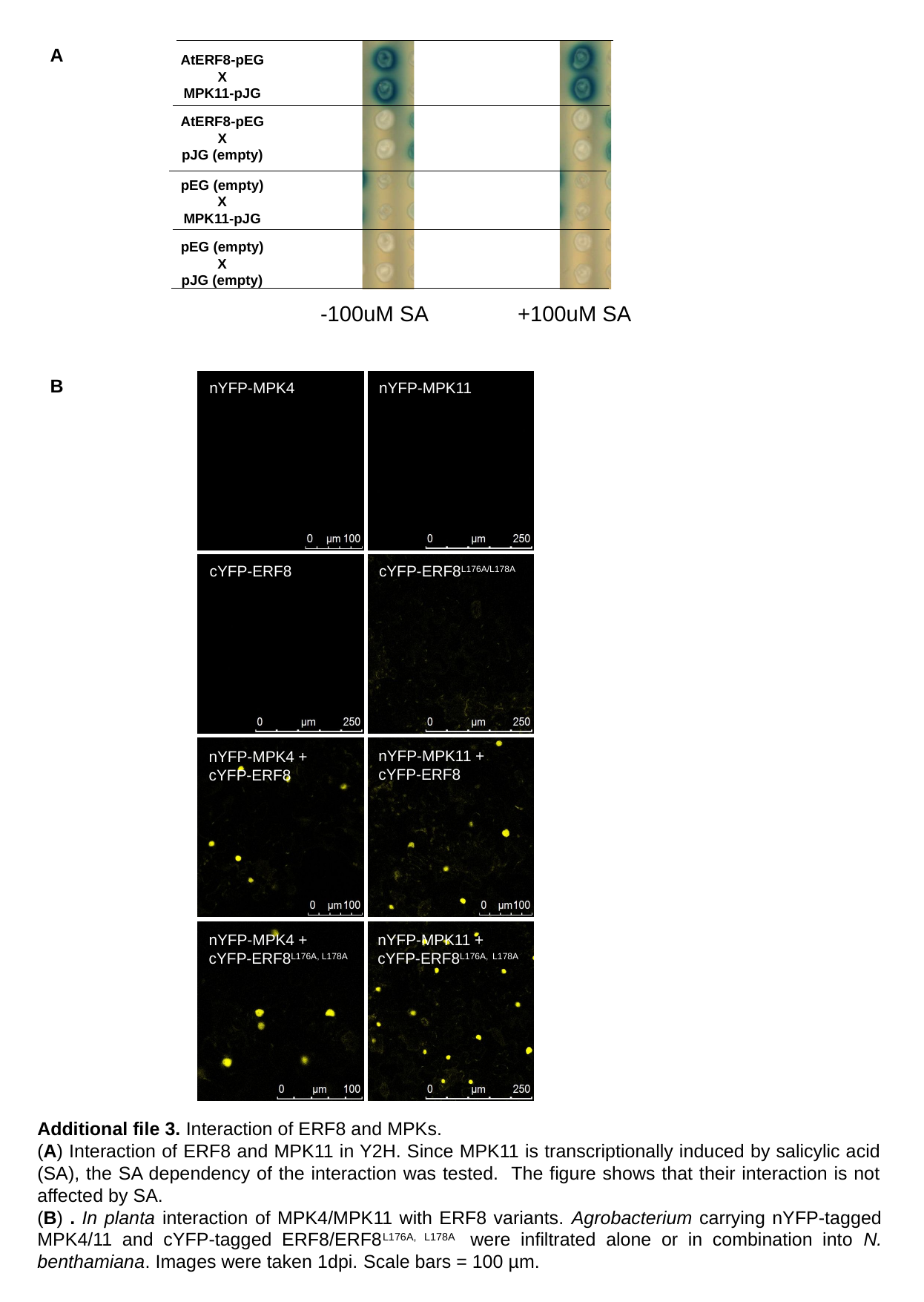

A
AtERF8-pEG
X
MPK11-pJG
AtERF8-pEG
X
pJG (empty)
pEG (empty)
X
MPK11-pJG
pEG (empty)
X
pJG (empty)
-100uM SA
+100uM SA
B
nYFP-MPK11
nYFP-MPK4
cYFP-ERF8
cYFP-ERF8L176A/L178A
nYFP-MPK11 +
cYFP-ERF8
nYFP-MPK4 +
cYFP-ERF8
nYFP-MPK11 +
cYFP-ERF8L176A, L178A
nYFP-MPK4 +
cYFP-ERF8L176A, L178A
Additional file 3. Interaction of ERF8 and MPKs.
(A) Interaction of ERF8 and MPK11 in Y2H. Since MPK11 is transcriptionally induced by salicylic acid (SA), the SA dependency of the interaction was tested. The figure shows that their interaction is not affected by SA.
(B) . In planta interaction of MPK4/MPK11 with ERF8 variants. Agrobacterium carrying nYFP-tagged MPK4/11 and cYFP-tagged ERF8/ERF8L176A, L178A were infiltrated alone or in combination into N. benthamiana. Images were taken 1dpi. Scale bars = 100 µm.
