# Supplementary material for: Arabidopsis ETHYLENE RESPONSE FACTOR 8 (ERF8) has dual functions in ABA signaling and immunity
Source: BMC Plant Biol. 2018 Sep 27;18:211. doi: 10.1186/s12870-018-1402-6 (PMC6161326; doi:10.1186/s12870-018-1402-6)
Supplement: Supplementary file 4 — Figure S4. Phosphorylation of Ser103 does not affect phosphorylation of other ERF8 residues in vitro. (PPTX 204 kb) [file 12870_2018_1402_MOESM4_ESM.pptx]

## Slide 1
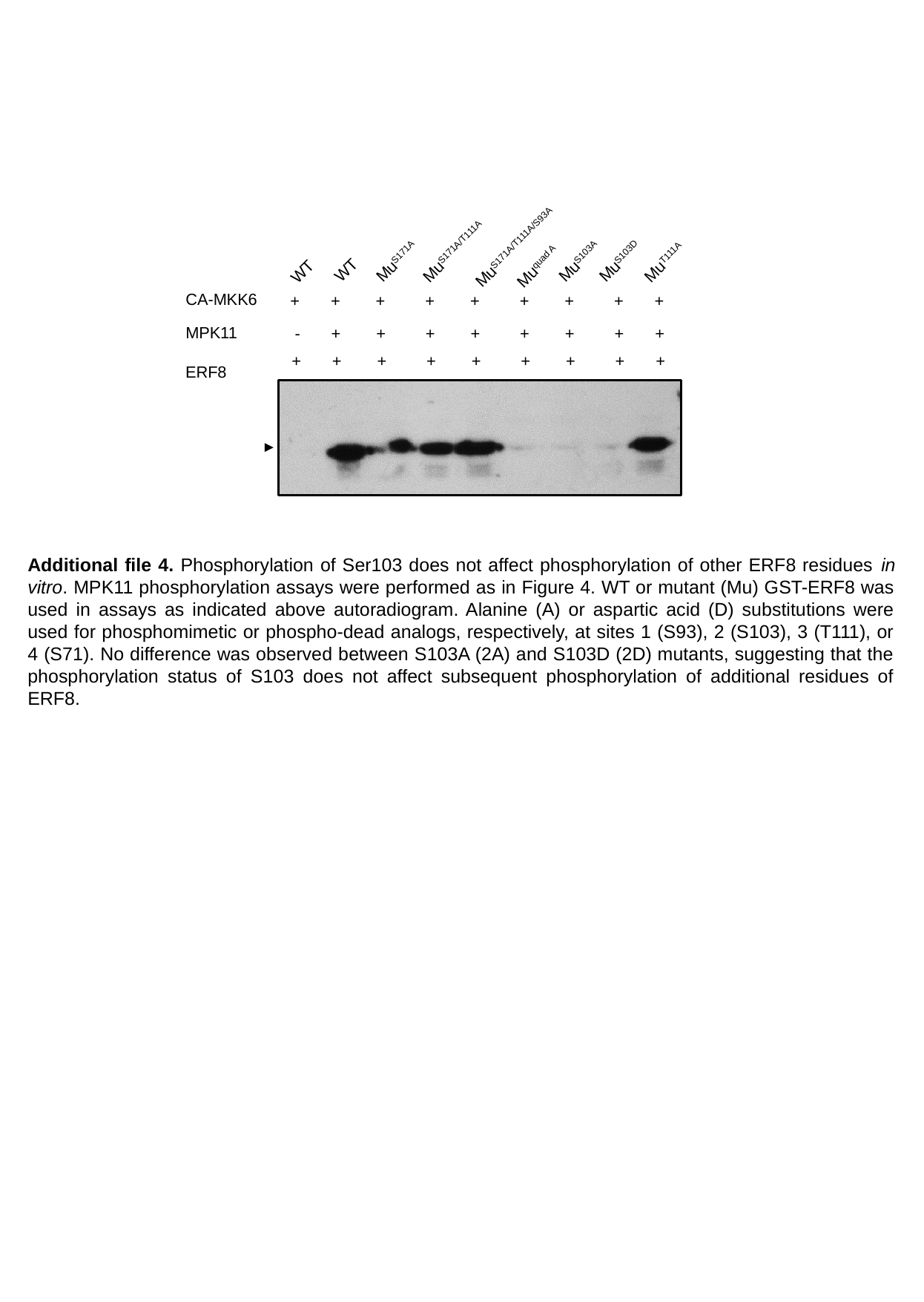

Muquad A
MuS171A/T111A/S93A
MuS171A/T111A
MuS103A
WT
MuS171A
MuS103D
MuT111A
WT
CA-MKK6
+ + + + + + + + +
MPK11
 - + + + + + + + +
 + + + + + + + + +
ERF8
Additional file 4. Phosphorylation of Ser103 does not affect phosphorylation of other ERF8 residues in vitro. MPK11 phosphorylation assays were performed as in Figure 4. WT or mutant (Mu) GST-ERF8 was used in assays as indicated above autoradiogram. Alanine (A) or aspartic acid (D) substitutions were used for phosphomimetic or phospho-dead analogs, respectively, at sites 1 (S93), 2 (S103), 3 (T111), or 4 (S71). No difference was observed between S103A (2A) and S103D (2D) mutants, suggesting that the phosphorylation status of S103 does not affect subsequent phosphorylation of additional residues of ERF8.
