# Supplementary material for: Arabidopsis ETHYLENE RESPONSE FACTOR 8 (ERF8) has dual functions in ABA signaling and immunity
Source: BMC Plant Biol. 2018 Sep 27;18:211. doi: 10.1186/s12870-018-1402-6 (PMC6161326; doi:10.1186/s12870-018-1402-6)
Supplement: Supplementary file 5 — Transient expression of ERF8 wt and variants in N. benthamiana. (PPTX 129 kb) [file 12870_2018_1402_MOESM5_ESM.pptx]

## Slide 1
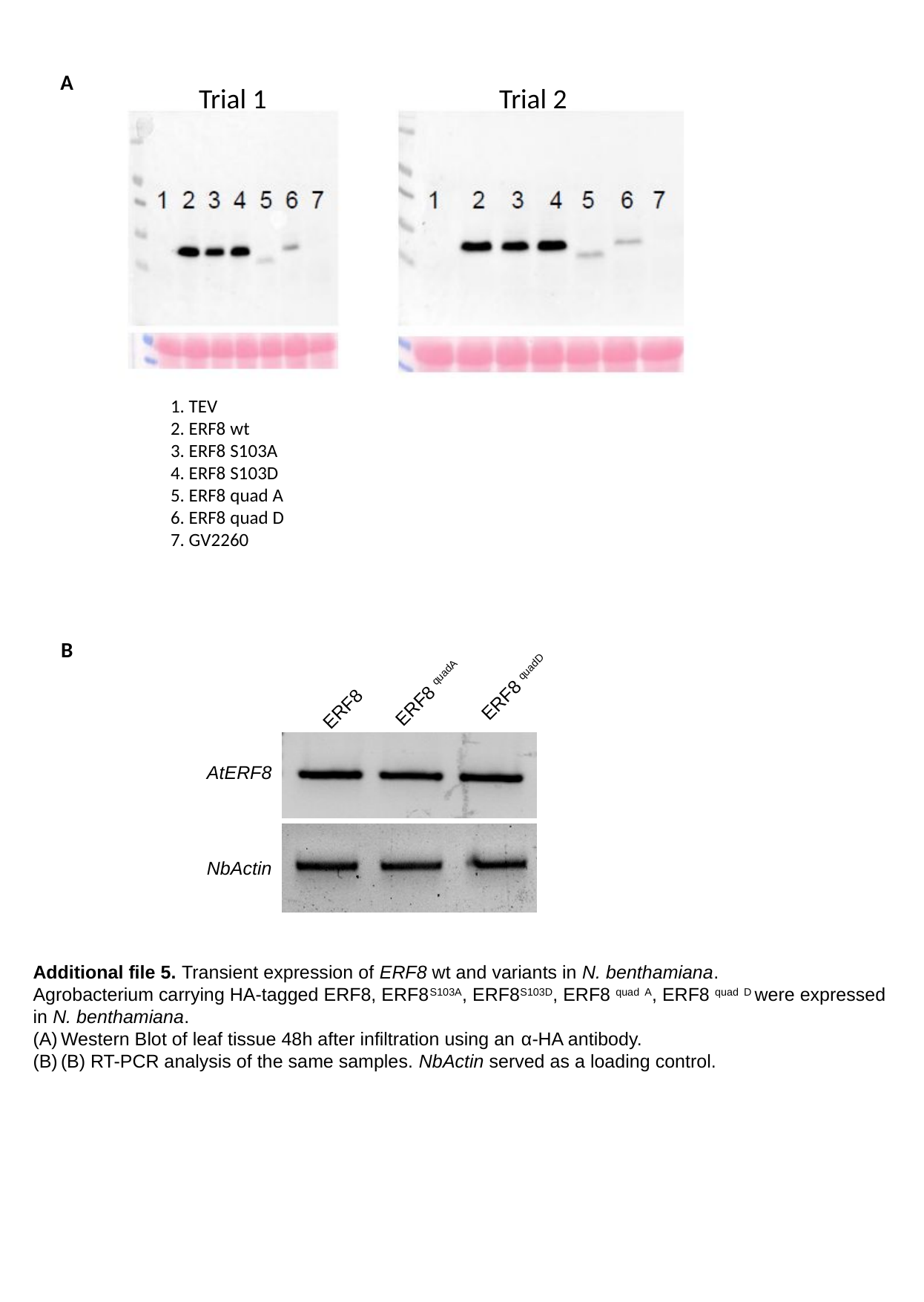

A
Trial 1 Trial 2
1. TEV
2. ERF8 wt
3. ERF8 S103A
4. ERF8 S103D
5. ERF8 quad A
6. ERF8 quad D
7. GV2260
ERF8 quadD
ERF8 quadA
B
ERF8
AtERF8
NbActin
Additional file 5. Transient expression of ERF8 wt and variants in N. benthamiana.
Agrobacterium carrying HA-tagged ERF8, ERF8S103A, ERF8S103D, ERF8 quad A, ERF8 quad D were expressed in N. benthamiana.
Western Blot of leaf tissue 48h after infiltration using an α-HA antibody.
(B) RT-PCR analysis of the same samples. NbActin served as a loading control.
