# Supplementary material for: Arabidopsis ETHYLENE RESPONSE FACTOR 8 (ERF8) has dual functions in ABA signaling and immunity
Source: BMC Plant Biol. 2018 Sep 27;18:211. doi: 10.1186/s12870-018-1402-6 (PMC6161326; doi:10.1186/s12870-018-1402-6)
Supplement: Supplementary file 6 — Figure S6. Analysis of ERF8 and its phosphorylation status under various conditions. (PPTX 154 kb) [file 12870_2018_1402_MOESM6_ESM.pptx]

## Slide 1
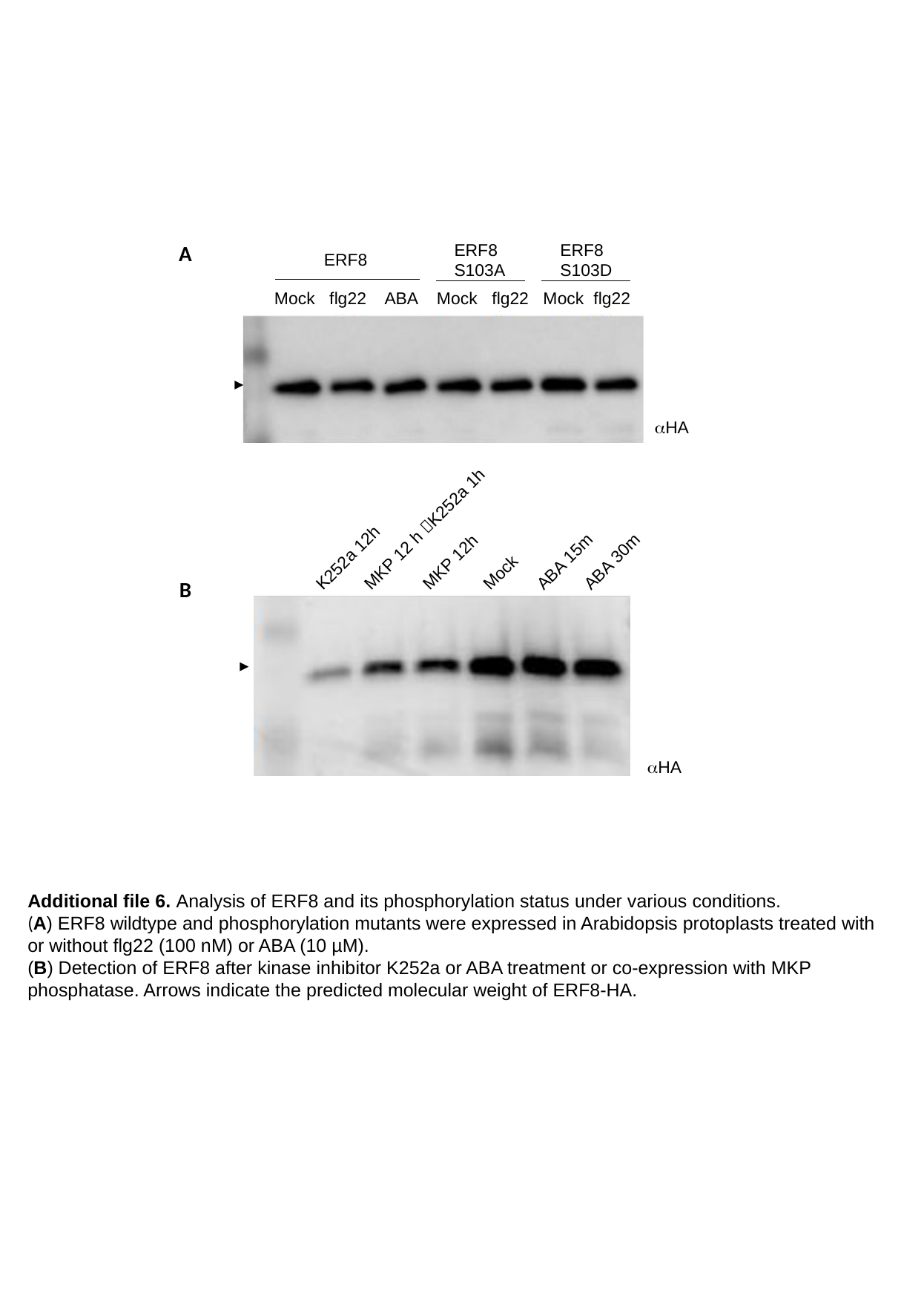

ERF8
S103A
ERF8
S103D
A
ERF8
Mock flg22 ABA Mock flg22 Mock flg22
αHA
MKP 12 h K252a 1h
ABA 30m
K252a 12h
ABA 15m
MKP 12h
Mock
B
αHA
Additional file 6. Analysis of ERF8 and its phosphorylation status under various conditions.
(A) ERF8 wildtype and phosphorylation mutants were expressed in Arabidopsis protoplasts treated with or without flg22 (100 nM) or ABA (10 µM).
(B) Detection of ERF8 after kinase inhibitor K252a or ABA treatment or co-expression with MKP phosphatase. Arrows indicate the predicted molecular weight of ERF8-HA.
