# Supplementary material for: Arabidopsis ETHYLENE RESPONSE FACTOR 8 (ERF8) has dual functions in ABA signaling and immunity
Source: BMC Plant Biol. 2018 Sep 27;18:211. doi: 10.1186/s12870-018-1402-6 (PMC6161326; doi:10.1186/s12870-018-1402-6)
Supplement: Supplementary file 8 — Figure S8. Analysis of differentially expressed genes. (PPTX 39 kb) [file 12870_2018_1402_MOESM8_ESM.pptx]

## Slide 1
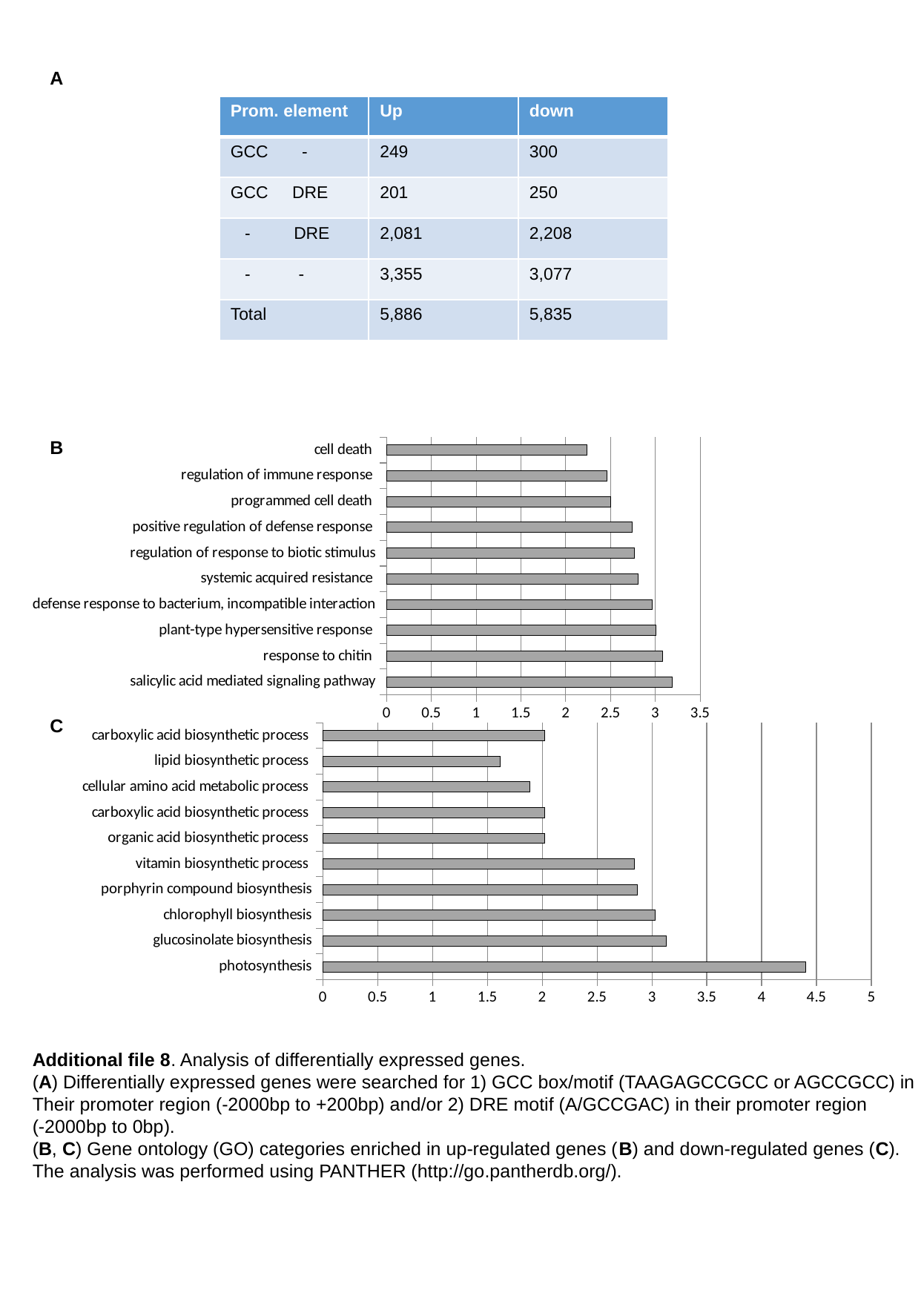

A
| Prom. element | Up | down |
| --- | --- | --- |
| GCC - | 249 | 300 |
| GCC DRE | 201 | 250 |
| - DRE | 2,081 | 2,208 |
| - - | 3,355 | 3,077 |
| Total | 5,886 | 5,835 |
B
### Chart
| Category | |
|---|---|
| salicylic acid mediated signaling pathway | 3.19 |
| response to chitin | 3.08 |
| plant-type hypersensitive response | 3.01 |
| defense response to bacterium, incompatible interaction | 2.97 |
| systemic acquired resistance | 2.81 |
| regulation of response to biotic stimulus | 2.77 |
| positive regulation of defense response | 2.74 |
| programmed cell death | 2.5 |
| regulation of immune response | 2.46 |
| cell death | 2.24 |C
### Chart
| Category | |
|---|---|
| photosynthesis | 4.4 |
| glucosinolate biosynthesis | 3.13 |
| chlorophyll biosynthesis | 3.03 |
| porphyrin compound biosynthesis | 2.87 |
| vitamin biosynthetic process | 2.84 |
| organic acid biosynthetic process | 2.02 |
| carboxylic acid biosynthetic process | 2.02 |
| cellular amino acid metabolic process | 1.89 |
| lipid biosynthetic process | 1.62 |
| carboxylic acid biosynthetic process | 2.02 |Additional file 8. Analysis of differentially expressed genes.
(A) Differentially expressed genes were searched for 1) GCC box/motif (TAAGAGCCGCC or AGCCGCC) in
Their promoter region (-2000bp to +200bp) and/or 2) DRE motif (A/GCCGAC) in their promoter region
(-2000bp to 0bp).
(B, C) Gene ontology (GO) categories enriched in up-regulated genes (B) and down-regulated genes (C).
The analysis was performed using PANTHER (http://go.pantherdb.org/).
